# The Effects of Spatial Scale on Breakdown of Leaves in a Tropical Watershed

**DOI:** 10.1371/journal.pone.0097072

**Published:** 2014-05-08

**Authors:** Renan S. Rezende, Mauricio M. Petrucio, José F. Gonçalves

**Affiliations:** 1 Graduate Program in Ecology of Department of Ecology and Zoology, Institute of Biological Sciences, Federal University of Santa Catarina, Florianópolis, Santa Catarina, Brazil; 2 Department of Ecology, Institute of Biology, University of Brasília, Brasília, Federal District, Brazil; University of Tasmania, Australia

## Abstract

The objective was to assess the effects of natural variation in the physical structure of the environment on biological communities and on the processing of *Eucalyptus cloeziana* and *Inga laurina* and to identify the controlling factors at different scales along stream order gradients. The study area consisted of 14 sampling sites distributed within a tropical watershed (1st, 2nd, 3rd and 4th order streams replicated in 4 sub-basins). Our samples consisted of 3 g of leaves of *E. cloeziana* (high-quality) and *I. laurina* (low-quality) placed in 252 bags with 10mm mesh (measured by the chemical composition of the detritus). Four samples of each leaf type were collected periodically (three times) over a period of 75–125 days and washed on a sieve to separate the invertebrates. A series of leaf disks were cut to determine ash-free dry mass, polyphenol, lignin, cellulose, total microbial biomass and fungal biomass, and the remaining material was oven-dried to determine the dry weight. We performed analyses within and between spatial scales (regional and local) to assess which watershed scale was the more import determinant of the leaf breakdown rate (*k*). The microbial and shredder were most influenced at the local scale (stream order). Shredders were influenced by microorganisms, with stronger interactions between them than were found to drive the *k* at the local scale. Moreover, differences in the overall *k* and abiotic variables were more strongly influenced at the regional scale (sub-basin), showing that the study scale alters the response of the studied variables. We found higher *k* values at higher values of water velocity, dissolved oxygen and temperature, all of which accelerate biological metabolism in response to variations on the regional scale. Watersheds with warmer microclimates and streams with higher nutrient levels and oxygen could be accelerating the ecosystem metabolism, independent of the detritus quality.

## Introduction

The characteristics of lotic ecosystems show natural patterns along an upstream-downstream gradient due to variations in geomorphology and topography in the watershed [1], [2]. Natural changes along this gradient (upstream to downstream) include an increase in the dimensions of the stream (width), changes (increases or decreases) in the velocity of the water, and openings in the riparian canopy that allow greater light penetration [3], [4]. Greater luminosity increases the temperature and accelerates photosynthetic production and autotrophic metabolism [3]. Along this gradient, therefore, the relative abundance of micro-organisms increases but that of invertebrates decreases (primarily shredders of organic matter), decreases from the headwaters to the downstream sections [1]. These natural changes also modify the energy input and cycling of organic matter in space and time [1], [5]. Senescent leaves are an important source of nutrients and food resources in heterotrophic metabolic environments, primarily in headwaters and small streams [6], [7]. However, this material is mineralized and available for use by primary producers and other trophic levels after its decomposition. As a result, leaf breakdown is a key process in lotic ecosystems [6], [8]. Leaf breakdown can be influenced by many factors, such as physical and chemical variables (associated with water and detritus) and the activities of communities of decomposers (micro-organisms and aquatic invertebrates) [6]–[13].

The study of leaf breakdown at the scale of a watershed allows us to observe emerging patterns and identify certain factors that structure the ecosystems at different scales [14], [15]. It is evident that a series of successively smaller and nested geomorphologic units can have various patterns and structures depending on the scale that is being analyzed [14], [16]. These patterns and structures can be observed in riffles and pools within continuous stretches, which are nested within large rivers that make up a watershed [15], [17], [18]. Studies that address only one scale are subject to problems because certain variables are measured directly in small areas or across short time intervals, whereas few can be measured at fine resolution over large areas [17], [18]. In addition, changes at smaller scales are not maintained at larger scales [14]. Therefore, the issue is that unless patterns are consistent at all scales, the findings at one scale cannot be extrapolated to yield accurate predictions at other scales. Accordingly, tests at multiple scales are needed for confident extrapolation. From this perspective, the evaluation of leaf breakdown at different scales enables the development of an integrated vision of the landscape during this important ecological process [19], [20].

The streams in a watershed can be considered within a hierarchical framework that presents organized view of spatial and temporal variations among and within stream systems along the “riverscapes” [21]–[23]. Therefore, several studies have examined leaf processing at large spatial scales across biomes [24], [25], latitudinal gradients [24], and altitudinal gradients [2] and influences of land use [26], [27]. Moreover, several recurrent topics emerge from considerations of several spatial scales. These topics include the relative importance of fungi and invertebrates [28], the use of bioindicators [20] and the hierarchical nature of lotic ecosystems [15]. Studies assessing allochthonous leaf breakdown at a watershed scale are rare worldwide, but they have been performed in temperate systems [15], [20], [23], [29].

In tropical streams, individual riffles or short stream reaches continue to be the most frequent sites for studies of leaf processing based on the traditional conceptual model [30], [31]. Several factors are known to cause variation in the rates of processing within and among tropical stream reaches [13], [32]. These factors include the effects of species mixing [33], [34], litter quality [35], [36], micro-organism communities [37], [38], invertebrate communities [39]–[41], detritivores and shredders [10], [42] and seasonal effects [8], [43], [44]. However, systematic assessments of variability in allochthonous leaf breakdown rates across multiple spatial scales using the watershed as the sampling unit have not been performed in tropical stream systems. This study could help to answer important questions, such as “how does spatial structure influence ecosystem function and how do we integrate within and between spatial scales to assess function”, suggested by Sutherland et al. [45] as one of 100 fundamental ecological questions.

Based on the premise that leaf breakdown is the result of the activity of decomposer organisms and the physical and chemical processes occurring in the stream water, which vary along the scale investigated in the study scale [6], [12], [13], the following hypotheses were tested in this study: (i) natural differences in the physical nature of the stream (increasing canopy opening, water velocity, temperature and nutrient concentrations) accelerate biological metabolism and leaf breakdown from upstream to downstream; (ii) shredders decrease and micro-organisms increase in importance from upstream to downstream; and (iii) differences in the overall *k* values will be more clearly understandable (strongly explained) at an increased spatial scale. The objective of the study was to assess the natural effects of variation in the physical environment on biological communities and the leaf breakdown rates of *Eucalyptus cloeziana F. Muell* and *Inga laurina Sw. Willd* and to identify the controlling factors at different scales along the stream order gradient.

## Methods

### The Study System

**Figure 1:**
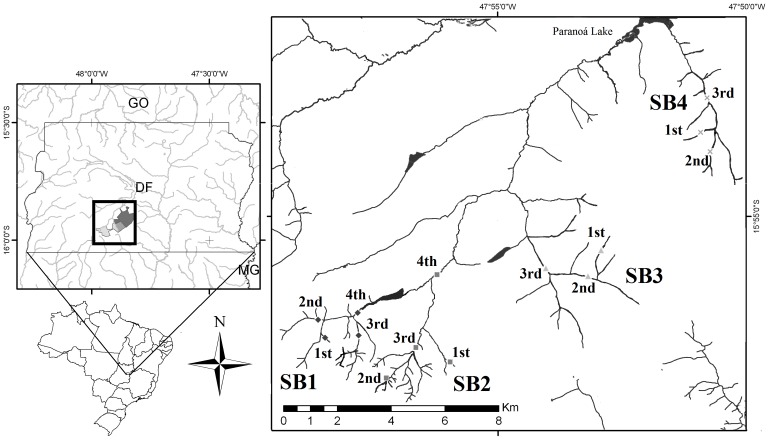
Sampling sites. Geographic location of the sampling sites consisting of streams of the 1^st^, 2^nd^, 3^rd^ and 4^th^ orders replicated in 4 sub-basins in the Gama-Cabeça de Veado watershed, Federal District (Brazil).

The study area consisted of 14 sampling sites distributed along the Gama-Cabeça do Veado watershed, a part of the Federal District in west central Brazil, comprising 1^st^, 2^nd^, 3^rd^ and 4^th^ order streams replicated in 4 sub-basins (Figure 1). The area includes important waterways that form the Paraná basin, a part of the Cerrado biome (Brazilian Savannah). The climate is tropical and has distinct rainy-hot (October to April) and cold-dry seasons (May to September). The mean annual temperature is 20°C, and the altitude varies between 1025 and 1150 m above sea level. The study area included three conservation units protecting the entire watershed and all sampling sites studied (Ecological Station of the University of Brasília, Ecological Reserve of IBGE and Ecological Station of the Botanical Gardens of Brasília). The study was approved by Ministry of Environment of Brazil through the System of Information and Authorization on Biodiversity (SISBIO) for activities with scientific purpose (code: 39629-1), and also was approved by the Scientific and Technical Council of the Ecological Station of the University of Brasília (code: 05–12), IBGE Ecological Reserve (code: 54 PC - PAD 1) and Botanical Gardens of Brasília (code: 13/2011).

### Procedures

The experiment was conducted from June through October 2011 (the winter season), a period of zero rainfall, resulting in a high level of homogeneity in the physical and chemical features of the environment. Based on their chemical composition, we selected leaves from two species for use as detritus. The leaves of an exotic species (*Eucalyptus cloeziana F. Muell*) were used to represent high-quality detritus. The planting of *Eucalyptus* monocultures in place of the native vegetation has potential repercussions for stream basins. The area in which the study was conducted does not contain *Eucalyptus* plantations. However, the substitution of this monoculture for native vegetation has occurred in neighboring basins, where this monoculture has expanded, as it has throughout Brazil, e.g., to supply charcoal for steelmaking and pulp for papermaking [35]. The low-quality detritus from a native species (abundant in riparian vegetation; *Inga laurina Sw. Willd*) was also used in the study, as this species best represents the plants of the Cerrado.

The leave The two types of leaves were chemically characterized by the mean values of total polyphenols (22.80 ± 2.5; 18.29 ± 1.8 mg/g^−1^), total tannic acids (0.003 ± 0.0002; 0.002 ± 0.0004 mg/g^−1^), lignin (42.61 ± 0.7; 45.94 ± 0.5%), cellulose (24.69 ± 1.5; 37.39 ± 1.2%), hardness (0.17 ± 0.1; 0.6 ± 0.3 cm/g^−1^), nitrogen (13.16 ± 1.3; 16.41 ± 1.0 g/kg^−1^) and phosphorus (0.46 ± 0.05; 0.53 ± 0.07 g/kg^−1^) in *E. cloeziana* and *I. laurina*, respectively. The breakdown rates for these two leaf types (collected in nets 1 m^2^ in area placed 1.5 m from the ground) were measured individually by the loss of weight of 3 g (± 0.1 g dry weight) of leaves, correcting for the initial humidity and transport loss [46], incubated in litter bags (15 × 15 cm, 10 mm mesh size).

In total, 252 litter bags were placed at a depth of 0.3 m in pool areas at the 14 sampling sites in 1^st^, 2^nd^, 3^rd^ and 4^th^ order streams [47] in the 4 sub-basins (Gama – SB1, Capetinga – SB2, Taquaras – SB3 and Cabeça de Veado – SB4 sub-basins). The removal of the *E. cloeziana* and *I. laurina* leaves occurred initially after 10 days of incubation, which corresponds to the expected time required for leaching and initiating microbial colonization [46]. After this sampling, the principal leaf breakdown rate (*k*) was used to estimate the next sampling time for each detritus type (at approximately 75% of the remaining mass), which was determined to be 40 days for *E. cloeziana* and 85 days for *I. laurina.* This stage of decomposition occurs when microbial activity is high and the invertebrate community is already established. Subsequently, the *k* value was used a second time to perform corrections and acquire new values to obtain approximately 50% of the remaining mass for each detritus type. This sampling time was determined to be 75 days for *E. cloeziana* and 125 days for *I. laurina*. After this period, the community established during the ecological process of degradative succession is affected by the reduction in the available detritus (additional information about k, see also Chapter 6 of [46]).

The sampling times were calculated by dividing the initial weight (W0) by the estimated value of *k*. This calculation yields the time for the total course of leaf processing (TLP, days). From the equation *W0/k  =  TLP*, we can calculate how many days will be required to reach a desired percentage of the initial weight (Wt). The first sample was collected after 10 days of incubation for both species, so that TLP for 10 days/0.25  =  day on which Wt  =  75%. The next sample was collected after 40 days for *E. cloeziana* and after 75 days for *I. laurina*, so that TLP 40/75 days/0.5  =  day by which Wt  =  50%. The above procedure was performed for each sample site (based on the mean value) and type of detritus. However, it was not possible to determine the final value for *I. laurina* because the dry season ended after 120 days, before 50% of the mass had been lost. Measurements after the end of the dry season would not have been meaningful because variations in rainfall and associated variations in other physical and chemical conditions would have influenced the results.

On removal from the streams, the litter bags were placed individually into insulated plastic bags and transported in thermal containers (± 4°C) to the laboratory. Temperature, electrical conductivity, pH, dissolved oxygen and water turbidity were obtained *in situ* with a multi-analyzer measured each time leaf bags were removed. The depth and average speed of the right, left and central portions of the watercourse were measured with a flow-meter, and the instantaneous discharge of water was then calculated. We collected 1 L of water to determine the nitrate [48], ammonia [49] and orthophosphate [50] concentrations. The canopy openings were quantified using hemispherical photographs taken with a digital camera equipped with a fish-eye lens. These photographs were later analyzed using Gap Light Analyzer software (2.0). The leaves were washed with tap water in a 120 µm mesh sieve. The invertebrates retained on the sieve were preserved in 70% alcohol for later identification and counting [51], [52]. The numbers of *taxa* and individuals were calculated for the aquatic invertebrate community, and biomass was obtained by desiccation at 60°C for 72 h. The invertebrates were classified into five feeding categories [51]–[53]: gathering-collectors (G–C), filtering-collectors (F–C), shredders (Sh), scrapers (Sc) and predators (P).

Five leaves from each sample were randomly collected, and three disks (1.2 cm diameter) were extracted from each leaf, resulting in three five-disk sets. One set was used to determine the remaining ash-free dry mass (AFDM; calculated after incineration in a muffle furnace at 550°C for 4 h), and the other sets were used to assess the ergosterol and ATP concentrations. The remaining material was oven-dried at 60°C for 72 h to determine its dry weight. The leaf breakdown rates (*k*) were calculated using the negative exponential model of percent mass lost over time (*W_t_  = W_0_e^−kt^*; Wt  =  remaining weight; W0  =  initial weight; −k  =  decay rate; t  =  time). After the leaves had been dried and weighed, they were pulverized for further analysis of the total polyphenol and tannic acid concentration [54], lignin and cellulose contents [55] and the resistance of leaves to rupture (hardness of intact leaves [46]). Values for total nitrogen were obtained using a CHN basic analyzer (Carlo Erba 1500 for WI; Thermo Electron Corp. Milan, Italy), and values for total phosphorus were obtained using the ascorbic acid method after acid digestion. The total micro-organism biomass was measured by quantifying ATP [56]. The biomass of aquatic Hyphomycetes was evaluated by quantifying ergosterol, a lipid exclusive to fungal membranes in this community [57].

### Data Analysis

An analysis of variance (function lm, package stats for R version 2.12.1; [58]) was used to analyze the physical and chemical parameters of the water (temperature, electrical conductivity, pH, dissolved oxygen, turbidity, nitrites, nitrates, orthophosphates and mean velocity) and the structure of stream stretches (instantaneous discharge of the stream and canopy openings in riparian vegetation) as dependent variables, using two categorical factors, namely, sub-basins and stream order. Stream order was also used as a co-variate (continuous variable). We also used the leaf mass remaining, invertebrate communities (number of *taxa*, density and biomass), the relative abundance of functional trophic groups of invertebrates (gathering-collectors, filtering-collectors, shredders, scrapers and predators) and microbial biomass (ATP and ergosterol) as dependent variables against the same two categorical factors. Stream order was also used as a co-variate (continuous variable). This procedure was performed similarly for both types of detritus. All models used a Gaussian distribution (link  =  log; test  =  F). We used an analysis of contrasts to discriminate among categorical variables. The normality of the data was tested using a Kolmogorov-Smirnov test, the homogeneity of variance was determined with a Levene test, and the data were transformed whenever necessary with the Naperian logarithm (ln) to obtain the best fit [58].

## Results

### Abiotic Variables

**Figure 2:**
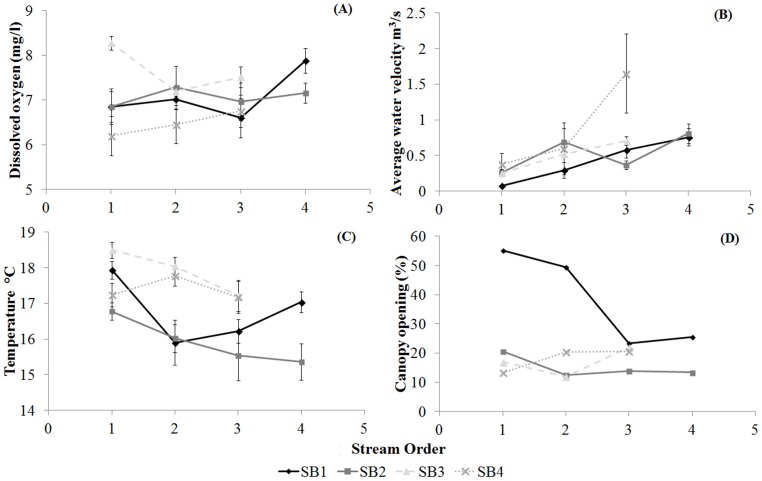
Abiotic variables. Mean values and standard errors for dissolved oxygen (A), water velocity (B), water temperature (C) and canopy opening (D) for the stream orders and among sub-basins.

The values of instantaneous discharge, electrical conductivity and nitrates were the highest in the 3^rd^ and 4^th^ order streams. In contrast, the 1^st^ order streams had the highest values for temperature, canopy opening and nitrite concentrations in the water, and the water velocity was the lowest. Dissolved oxygen, pH, turbidity and orthophosphates did not differ among the stream orders (analysis of contrasts, p < 0.05; Table S1, Table 1, Figure 2). In sub-basin 2 (SB2), we observed high electrical conductivity, high nitrite and nitrate concentrations and low water temperatures. The values for canopy opening and water velocity were highest in sub-basins 1 (SB1) and 4 (SB4), respectively. Dissolved oxygen and orthophosphates were highest in sub-basin 3 (SB3). Instantaneous discharge, pH and turbidity did not differ among the sub-basins (analysis of contrasts, p < 0.05; Table S1, Figure 2). We observed that the higher percentages of sums of squares and variance in instantaneous discharge and water velocity could be explained by differences in the stream order. However, dissolved oxygen, electrical conductivity, temperature, pH, turbidity, canopy opening, nitrates, nitrites and orthophosphates exhibited a high level of variance among the sub-basins (Table 1).

**Table 1:** Degrees of freedom (DF), residuals, sums of squares (%), F tests and analyses of variance and contrasts (AC) for dissolved oxygen, electrical conductivity, water temperature, pH, turbidity, water velocity, canopy openness and nitrites, nitrates and orthophosphates in sub-basins and along the stream orders in the Gama-Cabeça de Veado Basin.

		Df	Sum Sq (%)	F value	Pr(>F)	AC
Instantaneous discharge	Sub-Basin	3	5.71	1.97	0.127	
	Order	1	31.56	32.71	**<0.001**	1^st^ = 2^nd^ < 3^rd^ = 4^th^
	Residuals	65	62.72			
Dissolved oxygen	Sub-Basin	3	11.34	2.80	**0.047**	SB4 = SB2 = SB1 < SB3
	Order	1	0.87	0.65	0.425	
	Residuals	65	87.78			
Electrical conductivity	Sub-Basin	3	42.63	27.22	**<0.001**	SB4 = SB1 = SB3 < SB2
	Order	1	23.42	44.86	**<0.001**	1^st^ = 2^nd^ < 3^rd^ = 4^th^
	Residuals	65	33.93			
Temperature	Sub-Basin	3	22.29	6.66	**<0.001**	SB2 < SB1 = SB4 = SB3
	Order	1	5.15	4.62	**0.035**	4^th^ = 3^rd^ = 2^nd^ < 1^st^
	Residuals	65	72.55			
pH	Sub-Basin	3	5.44	1.25	0.298	
	Order	1	0.47	0.33	0.567	
	Residuals	65	94.07			
Turbidity	Sub-Basin	3	2.66	0.62	0.605	
	Order	1	4.23	2.96	0.090	
	Residuals	65	93.10			
Average water velocity	Sub-Basin	3	10.44	3.79	**0.014**	SB1 = SB3 = SB2 < SB4
	Order	1	29.77	32.37	**<0.001**	1^st^ < 2^nd^ = 4^th^ = 3^rd^
	Residuals	65	59.77			
Canopy opening	Sub-Basin	3	59.83	41.13	**<0.001**	SB2 = SB3 = SB4 < SB1
	Order	1	8.64	17.83	**<0.001**	4^th^ = 3^rd^ = 2^nd^ < 1^st^
	Residuals	65	31.52			
Nitrate	Sub-Basin	3	42.56	32.68	**<0.001**	SB3 = SB4 = SB1 < SB2
	Order	1	29.22	67.32	**<0.001**	1^st^ = 2^nd^ < 3^rd^ < 4^th^
	Residuals	65	28.21			
Nitrite	Sub-Basin	3	35.50	23.86	**<0.001**	SB4 = SB3 < SB1 < SB2
	Order	1	32.25	65.02	**<0.001**	4^th^ < 2^nd^ = 3^rd^ < 1^st^
	Residuals	65	32.24			
Orthophosphate	Sub-Basin	3	11.62	2.87	**0.043**	SB3 < SB1 = SB4 = SB2
	Order	1	0.54	0.40	0.527	
	Residuals	65	87.83			

### Leaf Breakdown Rates

**Figure 3:**
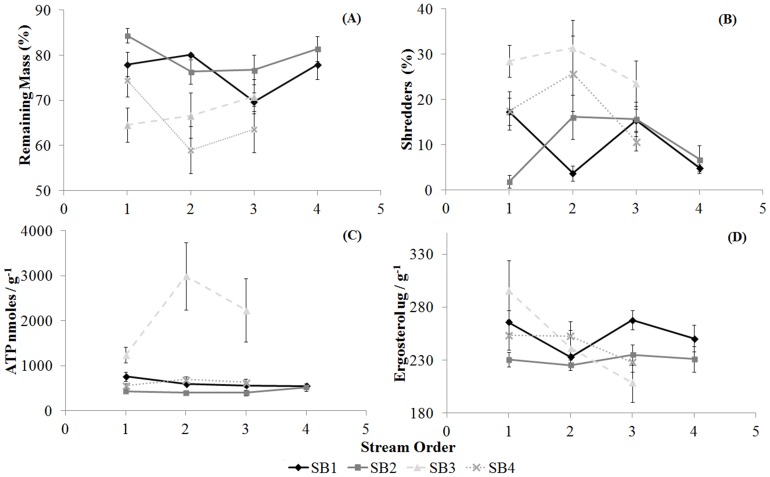
Leaf breakdown process in *E. cloeziana*. Mean values and standard errors for the remaining mass (A), shredder abundance (B), total microbial biomass (ATP; C) and fungal hyphomycetal biomass (Ergosterol; D) for the stream orders and sub-basins for *E. cloeziana*.

**Figure 4:**
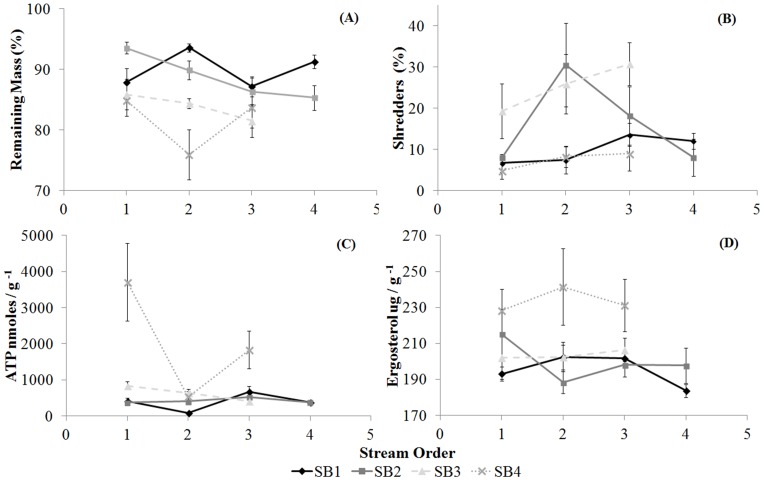
Leaf breakdown process in *I. laurina*. Mean values and standard errors for the remaining mass (A), shredder abundance (B), total microbial biomass (ATP; C) and fungal hyphomycetal biomass (Ergosterol; D) for the stream orders and sub-basins for *I. laurina*.

The leaf breakdown rates (*k*) were the highest in the 2^nd^ order streams, with values of −0.0083 and −0.0022 for *E. cloeziana* and *I. laurina*, respectively, followed by the 3^rd^ order stream sections, with values of −0.0071 and −0.0022. We also observed higher *k* values in 1^st^ order streams (−0.0053 and −0.0015) than in 4^th^ order streams (−0.0051 and −0.0018) for *E. cloeziana* and *I. laurina*, respectively. However, the remaining mass did not differ among stream orders for either of the detritus types (Figure S1, Table 2, Figure 3A and 4A). The highest *k* values were observed in SB4 (−0.0105 and −0.0030), followed by SB3 (−0.0088 and −0.0022 for *E. cloeziana* and *I. laurina*, respectively). For *E. cloeziana*, the values were −0.0062 and −0.0049, whereas *I. laurina* exhibited values of −0.0018 and −0.0016 (for SB1 and SB2, respectively). The remaining mass showed the lowest values in SB4 and SB3 among the sub-basins studied. The variance in the remaining mass was higher (by sums of squares) and also explained the variations in the sub-basins (Figure S1, Table 2, Figure 3A and 4A).

**Table 2:** Degrees of freedom (DF), residuals, sums of squares (%), F test and analyses of variance and contrasts (AC) for the remaining mass, density, richness and biomass of invertebrates, functional trophic groups of invertebrates (predators, shredders, gatherer-collectors, filtering-collectors and scrapers), ATP and ergosterol in sub-basins and along the stream orders in the Gama-Cabeça de Veado Basin for *E. cloeziana* and *I. laurina*.

				*E. cloeziana*					*I. laurina*		
		Df	Sum Sq (%)	F value	Pr(>F)	AC	Df	Sum Sq (%)	F value	Pr(>F)	AC
Remaining mass	Sub-Basin	3	15.05	6.51	**<0.001**	SB4 = SB3 < SB1 = SB2	3	11.01	4.72	**0.004**	SB4 = SB3 < SB2 = SB1
	Order	1	0.18	0.24	0.628		1	1.95	2.51	0.116	
	Residuals	110	84.76				112	87.04			
Invertebrate Density	Sub-Basin	3	0.63	0.25	0.864		3	9.29	4.14	**0.008**	SB2 = SB1 < SB3 = SB4
	Order	1	5.49	6.43	**0.013**	2^nd^ = 1^st^ = 3^rd^ < 4^th^	1	6.88	9.19	**0.003**	2^nd^ = 1^st^ = 3^rd^ < 4^th^
	Residuals	110	93.88				112	83.83			
Invertebrate Richness	Sub-Basin	3	12.42	5.65	**0.001**	SB2 = SB1 < SB4 = SB3	3	25.53	15.24	**<0.001**	SB2 < SB1 = SB3 = SB4
	Order	1	7.00	9.55	**0.003**	1^st^ = 4^th^ = 2^nd^ < 3^rd^	1	11.92	21.33	**<0.001**	1^st^ = 2^nd^ < 4^th^ = 3^rd^
	Residuals	110	80.58				112	62.56			
Invertebrate Biomass	Sub-Basin	3	1.29	0.48	0.697		3	4.10	1.62	0.189	
	Order	1	0.18	0.20	0.652		1	1.47	1.74	0.190	
	Residuals	110	98.53				112	94.43			
Predators	Sub-Basin	3	9.44	3.84	**0.012**	SB1 = SB2 = SB3 < SB4	3	11.87	5.09	**0.002**	SB1 = SB2 < SB3 = SB4
	Order	1	0.45	0.55	0.461		1	1.12	1.45	0.232	
	Residuals	110	90.11				112	87.01			
Shredders	Sub-Basin	3	13.89	5.99	**0.001**	SB2 = SB1 = SB4 < SB3	3	9.76	4.09	**0.008**	SB4 = SB1 = SB2 < SB3
	Order	1	1.12	1.45	0.232		1	1.26	1.58	0.211	
	Residuals	110	84.99				112	88.99			
Gatherer-Collectors	Sub-Basin	3	22.51	10.68	**<0.001**	SB4 = SB3 < SB2 = SB1	3	27.15	14.29	**<0.001**	SB3 = SB2 = SB4 < SB1
	Order	1	0.23	0.33	0.565		1	1.91	3.02	0.085	
	Residuals	110	77.26				112	70.93			
Filtering-Collectors	Sub-Basin	3	11.14	4.89	**0.003**	SB1 < SB3 = SB4 = SB2	3	10.61	4.75	**0.004**	SB1 = SB2 = SB3 < SB4
	Order	1	5.23	6.88	**0.010**	1^st^ = 2^nd^ = 4^th^ < 3^rd^	1	5.98	8.03	**0.005**	1^st^ < 4^th^ = 2^nd^ = 3^rd^
	Residuals	110	83.63				112	83.41			
Scrapers	Sub-Basin	3	21.70	10.21	**<0.001**	SB3 = SB4 = SB2 < SB1	3	11.79	5.07	**0.002**	SB3 = SB4 = SB2 < SB1
	Order	1	0.34	0.47	0.493		1	1.50	1.94	0.166	
	Residuals	110	77.96				112	86.71			
ATP	Sub-Basin	3	26.40	13.19	**<0.001**	SB2 = SB1 = SB4 < SB3	3	17.59	8.07	**<0.001**	SB2 = SB1 = SB3 < SB4
	Order	1	0.20	0.31	0.581		1	1.06	1.46	0.230	
	Residuals	110	73.40				112	81.35			
Ergosterol	Sub-Basin	3	2.25	0.87	0.457		3	9.82	4.07	**0.009**	SB1 = SB2 = SB3 < SB4
	Order	1	3.27	3.81	0.053		1	0.10	0.13	0.720	
	Residuals	110	94.47				112	90.08			

### Biotic Community

The density of invertebrates was higher in 4^th^ order streams for both detritus types (means of 27 and 21 ind/g for *E. cloeziana* and *I. laurina*, respectively). Differences for *I. laurina* were observed among sub-basins, with high values in SB3 (mean 24 ind/g) and SB4 (mean 26 ind/g). The number of *taxa* was significantly different among the stream orders and sub-basins, with the highest values in SB3 (mean of 7 and 6 *taxa* for *E. cloeziana* and *I. laurina*, respectively) and SB4 (mean of 6 *taxa* for *E. cloeziana* and *I. laurina*), primarily in 3^rd^ order streams for both detritus types (mean of 7 and 6 *taxa* for *E. cloeziana* and *I. laurina*, respectively). However, the biomass (total mean 0.003 and 0.002 ind/g for *E. cloeziana* and *I. laurina*, respectively) did not differ among the stream orders or sub-basins for either of the detritus types. The high variances in density, richness and biomass (in terms of the percentage of the sums of squares) were explained by differences in the sub-basins for both detritus types, except for the density of invertebrates in *I. laurina* (Figure S2 and S3, Table 2).

The functional trophic groups differed significantly among stream orders only for the filtering-collectors, with the highest values in the 3^rd^ order streams (mean 20% for *E. cloeziana* and *I. laurina*) and the lowest in the 1^st^ order streams (mean of 10% for *E. cloeziana* and *I. laurina*) for both detritus types. The relative abundance of predators was higher in SB3 (mean 24 and 25% for *E. cloeziana* and *I. laurina*, respectively) and SB4 (mean of 32 and 34% for *E. cloeziana* and *I. laurina*, respectively), whereas the values for shredders were higher in SB3 (mean of 27 and 25% for *E. cloeziana* and *I. laurina*, respectively) for both detritus types. However, SB1 exhibited high abundances of gathering-collectors (mean of 41 and 50% for *E. cloeziana* and *I. laurina*, respectively) and scrapers (mean of 18 and 16% for *E. cloeziana* and *I. laurina*, respectively) but a low abundance of filtering-collectors (mean of 6 and 8% for *E. cloeziana* and *I. laurina*, respectively) for both detritus types. The high variance in the relative abundance for all functional trophic groups (by the sums of squares) was also explained by changes in sub-basins for both detritus types (Figure S2 and S3, Table 2, Figure 3B and 4B).

The ATP values differed only among sub-basins for both detritus types, with the highest values in SB3 (mean of 2155.8 nmoles/g AFDM) for *E. cloeziana* (total mean 991.8 nmoles/g AFDM) and in SB3 (mean 633.3 nmoles/g AFDM) and SB4 (mean 2023.9 nmoles/g AFDM) for *I. laurina* (total mean 847.8 nmoles/g AFDM) (Table 2; Fig. 2C and 3C). There were no differences in the ergosterol concentrations among the hydrological stream orders and sub-basins for *E. cloeziana* (total mean 541.2 µg/g). However, we found higher ergosterol concentrations for *I. laurina* (total mean 382.9 µg/g) in SB3 (mean 392.6 µg/g) and SB4 (mean 464.1 µg/g), although they did not differ among the hydrological stream orders. The variances in ATP and ergosterol concentrations were also explained by changes in sub-basins for both detritus types, except for ergosterol in *E. cloeziana*, which showed a high level of variation with stream order (Figure S2 and S3, Table 2, Figure 3D and 4D).

## Discussion

### Scale Analysis

The instantaneous discharge, water velocity, turbidity and nitrogen series (nitrate and nitrite) were more influenced by changes in stream order (high heterogeneity), with higher values downstream (increasing from 1^st^ to 4^th^ order), as expected according to Vannote et al. [1], except that nitrites exhibited the inverse pattern. The finding of relatively few influences at local scales can be explained by the large discontinuities inherent in smaller geomorphological units (habitat patches create discontinuities in space) that increase the potential influence from the local characteristics of the environment [4], [5], [14], [18]. Flow changes, for example, create hydrological discontinuities along stream corridors and isolate habitats. However, the other abiotic variables were influenced by changes in sub-basins (high homogeneity) that correspond to regional scales [19], [21]. These factors worked at the watershed level and may increase its fragility in the face of intense climatic changes because the climate is the primary controlling factor at large scales [22]. The basins are influenced by environmental factors that systematically change across longitudinal (upstream/downstream), vertical (sediment/water) and lateral (terrestrial/aquatic) gradients, forming different spatial and temporal patterns at regional and local scales [5], [17], [18].

As expected, the high-quality detritus (*E. cloeziana*) showed more rapid leaf breakdown rates (*k*) than the low-quality (*I. laurina*) detritus, indicating that the rate could be driven by micro-scale processes [14]. These findings represent important evidence that the riparian vegetation could be responsible for determining the ecosystem characteristics [30], [31], [59], as also proposed by Gonçalves et al. [35] for tropical systems, highlighting the need to study this vegetation. For example, if riparian vegetation is composed of plant species that have a higher stoichiometric ratio (higher quality), we expect more rapid response for organic matter cycling (higher decomposition). This direct relationship between terrestrial and aquatic ecosystems demonstrates that any modification in a riparian ecosystem would affect the function, primarily in areas composed of palatable plants (high quality and decomposition), corroborating the work of Frauendorf et al. [60]. Thus, lower quality vegetation (slower decomposition) will be less sensitive to other factors, and this may explain the resistance of the Brazilian savanna in comparison with other Brazilian tropical systems [61]. Moreover, the leaf breakdown at the macro-scale was more influenced by the regional scale (sub-basin) than by the local scale (stream order) [14], confirming our hypothesis. This result indicates that the patterns observed in studies covering a given time period (timely studies), common in tropical literature (for more see also [13], [32]), cannot be generalized from local to regional scales [17], [29], [62] or to whole watersheds [14]–[16], [18]. In addition, this finding may indicate fragility in the upstream basins due to the slower leaf breakdown rates. However, the upstream area is a source of nutrients and organisms for the downstream basins [1]. The upstream basins can give support productivity and may be responsible for extending the depuration capability of the system (as represented by the microbial pools) and maintaining functionality downstream [60]. Therefore, we believe that the association between detritus quality (important at the micro-scale) and the environmental features of the watershed (important at the macro-scale) is responsible for shaping organic matter cycling in the watershed and should be further investigated in future studies.

Previous studies indicating that the microbial community is the principal decomposer [6], [30], [35], are confirmed by our results, as we found that the high-quality leaf species was also more susceptible to leaching and microbial action, whereas the low-quality leaf species was influenced primarily by fungal colonization. Both leaf samples were consumed by shredders, but a higher abundance of shredders was observed in the *E. cloeziana* detritus. We also found a higher loss of mass due to the high water velocity (mechanical fragmentation and leaching), dissolved oxygen and temperatures, which accelerate biological metabolism [37], [63]. The detritus quality is important only for defining the local rates and their pathways for leaf breakdown [6], [35], [64]. However, the detritus quality has little influence on the general pattern along the “riverscape” and at any specific scale [15]. Therefore, based on an analysis of the samples after a certain percentage of mass has been lost (25, 75 and 50%) and not simply at predefined time points (e.g., 7, 15, 30 days), it is possible to show a clear colonization effect independent of quality. To be sure, detritus quality is a highly important determinant of the abundance of shredders and explains the importance of shredders for both detritus types [39]. We cannot study the variations associated with spatial scale in terms only of the local context because there are many factors in the ecological levels (community and ecosystem) that are responsible for variability found in the large scale [18], [29]. However, the local approach has been used in all previous tropical studies of leaf breakdown [12], [13].

### Leaf Breakdown Rates

In agreement with the proposal of Gonçalves et al. [13] for tropical systems, the *k* values of *E. cloeziana* were classified as intermediate (−0.0173 > k > −0.0041), and those of *I. laurina* were classified as slow (k < −0.0041) for all sampling sites, indicating a strong influence of detritus quality. The high leaching due to the high solubility of polyphenols and tannins (secondary compounds) in *E. cloeziana* can accelerate the decay rate [64], [65]. Therefore, the rapid leaching of these secondary compounds, which has an inhibitory effect on detritivores, as well as the lower hardness of *E. cloeziana*, did not limit biotic colonization due to the low residence time in this type of detritus [35], [64]. Additionally, *Eucalyptus* sp. (an exotic species) is rapidly colonized and decomposed in the Brazilian savannah. It is possible that this pattern is due to the high quality of *Eucalyptus* relative to native species [35], [39], [66].

In contrast, we found lower breakdown rates for *I. laurina* which were most likely a consequence of a high content of structural compounds (lignin and cellulose) and relative hardness (cuticle thickness), hindering the release of other chemical compounds (e.g., polyphenols, nitrogen and phosphorus [35], [65], [66]). Therefore, the chemical characteristics of detritus determine the speed of processing (primarily at local scales), showing that leaf breakdown rates increase with quality and palatability [6], [7], [35], [39]. Detritus quality is of lower importance when if it is observed at different scales. In the study area, we observed that the regional scale is decisive for driving the general pattern of this important ecological process along the “riverscapes” [21], [22].

### Abiotic and Biotic Variables in Leaf Breakdown

The natural environmental changes that occur across stream orders [4] were not sufficient to modify the remaining mass, and the local scale could not affect the decomposer communities (shredders and microorganisms) for either of the detritus types. Decomposer communities are the driving factors for leaf breakdown, and their absence leads to similar breakdown patterns along the stream order gradient [6]. This finding might indicate that ecological functioning in headwater streams (1^st^ to 3^rd^ order) was similar within the same sub-basin [1]. However, this process might change over a large spatial gradient, as represented by the sub-basin scale [22]. Nevertheless, increases in richness and the density of invertebrates and a decrease in the abundance of filtering-collectors for both detritus types were observed across this large spatial gradient. These results demonstrate that these variables had no effect on leaf breakdown [52], [53].

The values of the remaining mass for both detritus types were lower in SB4 and SB3 (high decomposition) than in the other sub-basins. It is probable that the reason for this difference was the higher temperatures, dissolved oxygen concentrations and water velocities resulting from the microclimate of the geographic location (within a valley). Therefore, the higher temperatures [63] and oxygen concentrations [37] observed in SB4 and SB3 may elevate the metabolic activity of the decomposer community [9], especially microorganisms. The higher metabolic activity of the decomposer community, associated with high water velocity (mechanical fragmentation and leaching), which increased the degree of physical abrasion [67], accelerated the leaf breakdown rates. In SB4 and SB3, higher density and richness of invertebrates and higher shredder abundance, with the greatest densities in *E. cloeziana*, were also observed. The shredders directly utilize leaf tissues for feeding, and increasing biological fragmentation [7], [42] can also accelerate the leaf breakdown rates [26]. Certain shredders in these locations (genus *Phylloicus*) can build their capsules from leaf tissue, and this use of leaf material also contributes to fragmentation [52], [53].

The relative abundance of shredders was influenced by variation, primarily among the sub-basins. A greater relative abundance of shredders in comparison with other tropical systems was observed in SB3 for both detritus types [10], [39], [42]. A low relative abundance of shredders was found in the other sub-basins. This result is consistent with the findings of previous studies in the Cerrado [13], [36], [39]. The importance of shredders for leaf breakdown is unclear in the tropics due to their low abundance or absence in these streams [10], [39], [42], but studies have shown little effect in tropical streams [11], [33]. From a global perspective, the strong effects observed in the current study were most likely due to the preference of shredders (primarily Trichoptera and Plecoptera) for high altitudes (due to the lower temperatures) in tropical regions [42]. The preference of this group for *E. cloeziana* indicates that detritus quality is also important [10] and that the composition of the vegetation influences the functioning of aquatic systems. Therefore, higher altitudes (low temperature [42]), high dissolved oxygen, the composition of the flora (ideally including *E. cloeziana* [10]) and moderate values of nutrient concentrations in the water [62] favor a high abundance of shredders. The predominance in the Cerrado of leaves that are low in nutrients [66], is associated with hydric and thermic stress and could be responsible for the absence or low abundance of shredders found in most tropical streams [10], [42].

The high-quality detritus (*E. cloeziana*) was shown to be most influenced by the total microbial community and the low-quality detritus (*I. laurina*) by the fungal community in SB4 and SB3 (high decomposition). *E. cloeziana* has elevated amounts of labile compounds, facilitating the activity of bacteria (rapid life cycles) that use compounds derived from the leaching of the leaves of labile detritus as their preferred resource [68]. These bacteria could be important during leaf breakdown and not only, as observed by several authors, at the early stages of the process [6], [13], [35]. However, this pattern is rarely observed in tropical streams. In lower-quality detritus (*I. laurina*), we observed an interaction between the biomass of the two microbial communities, highlighting fungi as the principal component [63]. Due to its high capacity to metabolize refractory molecules (e.g., cellulose and lignin) and to decompose them, the fungal community is the primary decomposer in tropical streams, and this principle explains the great significance of fungi in *I. laurina* decomposition [6], [35], [68]. Fungal action can increase the palatability of detritus, as well as its nutritional quality, for other decomposers, and the high biomass of fungi might be another factor responsible for the higher abundance of shredders in these sub-basins [7], [35].

In general, we conclude that variations in scale contribute to the variation in the leaf breakdown rate, highlighting the importance of similar studies of this type that determine effects at different scales. The variability of the physical structure of streams (primarily temperature, dissolved oxygen and nutrients) accelerates leaf breakdown from upstream to downstream, but this process was only demonstrated at the sub-basin scale in the location studied, partially corroborating the initial hypothesis. The replacement of shredder invertebrates by microorganisms was observed but was contrary to the prediction of our hypothesis. Shredders were favored by microorganisms (primarily in *E. cloeziana*), with stronger interactions between them than those previously found to drive leaf breakdown rates. Based on our interest in the influence of spatial structure on ecosystem functions, we observed that watersheds with warmer microclimates and streams with higher nutrient levels and oxygen in the water could be accelerating the metabolism of the ecosystem in the watershed, with increased negative effects downstream. For the management of tropical watersheds, we noted that the upstream areas are more fragile and sensitive to environmental impacts but show greater importance in the cycling of nutrients. We performed analyses within and between spatial scales to assess the relative importance of various watershed scales in determining the local breakdown rate for leaves. Local characteristics are responsible for the diversification of this process across the “riverscape”, and high heterogeneity underscores the difficulty of making predictions based on local studies.

## Supporting Information

Table S1 **Abiotic variables in sampling sites.** Average values and the standard deviation of outflow, dissolved oxygen in the water (mg l^−1^), electrical conductivity (µS-cm^2^), water temperature (Temp. °C), pH, turbidity (NTU), water velocity (m-s), nitrite, nitrate, orthophosphate (mg l^−1^) and the percentage of canopy openness (%) in sub-basin and stream order along the Gama-Cabeça de Veado Basin.
(DOCX)

Figure S1 **Remaining mass over time in sampling sites.** Percentages of remaining mass along of the day in *E. cloeziana* (A and C) and *I. laurina* (B and D), between stream order (A e B) and sub-basin (C and D).
(DOCX)

Figure S2 **Biotic Community over time in** ***E. cloeziana*** **detritus.** Average values and standard error of density (A and B), richness (C and D), biomass (E and F) of aquatic invertebrates, total microbial biomass (ATP; G and H) and fungal hyphomicetos biomass (I and J) along of the days in *E. cloeziana*, among stream order (A, C, E, G and I) and sub-basin (B, D, F, H and J).
(DOCX)

Figure S3 **Biotic Community over time in** ***I. laurina*** **detritus.** Average values and standard error of density (A and B), richness (C and D), biomass (E and F) of aquatic invertebrates, total microbial biomass (ATP; G and H) and fungal hyphomicetos biomass (I and J) along of the days in *I. laurina*, among stream order (A, C, E, G and I) and sub-basin (B, D, F, H and J).
(DOCX)
