# Supplementary material for: The Effects of Spatial Scale on Breakdown of Leaves in a Tropical Watershed
Source: PLoS One. 2014 May 8;9(5):e97072. doi: 10.1371/journal.pone.0097072 (PMC4014586; doi:10.1371/journal.pone.0097072)
Supplement: Table S1 — Abiotic variables in sampling sites. Average values and the standard deviation of outflow, dissolved oxygen in the water (mg l−1), electrical conductivity (µS-cm2), water temperature (Temp. °C), pH, turbidity (NTU), water velocity (m-s), nitrite, nitrate, orthophosphate (mg l−1) and the percentage of canopy openness (%) in sub-basin and stream order along the Gama-Cabeça de Veado Basin. (DOCX) [file pone.0097072.s001.docx]

Table S1. Average values and the standard deviation of outflow, dissolved oxygen in the water (mg l^-1^), electrical conductivity (µS-cm^2^), water temperature (Temp. °C), pH, turbidity (NTU), water velocity (m-s), nitrite, nitrate, orthophosphate (mg l^-1^) and the percentage of canopy openness (%) in sub-basin and stream order along the Gama-Cabeça de Veado Basin.

| Sub-basin | Order | Outflow | | | Oxygen | | | Conductivity | | | Temperature | | | pH | | | Turbidity | | | Velocity | | | Nitrite | | | Nitrate | | | Orthophosphate | | | Canopy openness |
| --- | --- | --- | --- | --- | --- | --- | --- | --- | --- | --- | --- | --- | --- | --- | --- | --- | --- | --- | --- | --- | --- | --- | --- | --- | --- | --- | --- | --- | --- | --- | --- | --- |
| SB1 | 1st | 0.02 | ± | 0.01 | 6.85 | ± | 0.49 | 4.24 | ± | 0.34 | 17.94 | ± | 0.36 | 5.55 | ± | 0.18 | 4.65 | ± | 0.92 | 0.08 | ± | 0.01 | 18.65 | ± | 0.56 | 0.43 | ± | 0.01 | 6.84 | ± | 0.13 | 55.09 |
| SB1 | 2nd | 0.11 | ± | 0.06 | 7.02 | ± | 0.33 | 3.55 | ± | 0.18 | 15.90 | ± | 0.90 | 6.00 | ± | 0.30 | 4.61 | ± | 0.53 | 0.29 | ± | 0.16 | 6.87 | ± | 0.56 | 0.13 | ± | 0.01 | 4.76 | ± | 0.13 | 49.50 |
| SB1 | 3rd | 0.48 | ± | 0.20 | 6.60 | ± | 0.62 | 4.01 | ± | 0.32 | 16.22 | ± | 0.47 | 5.82 | ± | 0.27 | 7.82 | ± | 3.06 | 0.58 | ± | 0.16 | 21.53 | ± | 0.37 | 0.09 | ± | 0.01 | 2.20 | ± | 0.00 | 23.40 |
| SB1 | 4th | 1.23 | ± | 0.44 | 7.88 | ± | 0.39 | 9.65 | ± | 0.19 | 17.04 | ± | 0.42 | 5.97 | ± | 0.18 | 4.86 | ± | 0.52 | 0.76 | ± | 0.17 | 61.58 | ± | 1.48 | 0.10 | ± | 0.01 | 3.52 | ± | 0.07 | 25.61 |
| SB2 | 1st | 0.03 | ± | 0.01 | 6.85 | ± | 0.57 | 10.25 | ± | 0.69 | 16.78 | ± | 0.34 | 5.81 | ± | 0.09 | 10.57 | ± | 2.84 | 0.26 | ± | 0.06 | 11.06 | ± | 0.74 | 0.73 | ± | 0.01 | 3.33 | ± | 0.20 | 20.50 |
| SB2 | 2nd | 0.31 | ± | 0.11 | 7.29 | ± | 0.68 | 6.05 | ± | 0.76 | 16.02 | ± | 0.55 | 6.22 | ± | 0.11 | 3.13 | ± | 0.45 | 0.69 | ± | 0.26 | 42.73 | ± | 0.37 | 0.19 | ± | 0.01 | 3.81 | ± | 0.07 | 12.53 |
| SB2 | 3rd | 0.22 | ± | 0.08 | 6.97 | ± | 0.59 | 16.73 | ± | 0.46 | 15.54 | ± | 1.01 | 6.09 | ± | 0.11 | 3.01 | ± | 0.71 | 0.37 | ± | 0.08 | 94.56 | ± | 0.37 | 0.31 | ± | 0.01 | 8.74 | ± | 0.60 | 13.86 |
| SB2 | 4th | 1.19 | ± | 0.26 | 7.16 | ± | 0.31 | 15.24 | ± | 0.79 | 15.36 | ± | 0.73 | 5.91 | ± | 0.16 | 2.54 | ± | 1.02 | 0.81 | ± | 0.19 | 148.21 | ± | 6.11 | 0.07 | ± | 0.01 | 2.58 | ± | 0.27 | 13.36 |
| SB3 | 1st | 0.11 | ± | 0.05 | 8.28 | ± | 0.21 | 2.70 | ± | 0.43 | 18.50 | ± | 0.32 | 6.03 | ± | 0.34 | 5.49 | ± | 0.43 | 0.25 | ± | 0.06 | 5.83 | ± | 0.37 | 0.05 | ± | 0.01 | 1.82 | ± | 0.01 | 16.93 |
| SB3 | 2nd | 0.29 | ± | 0.07 | 7.20 | ± | 0.16 | 7.91 | ± | 0.73 | 18.04 | ± | 0.37 | 5.75 | ± | 0.25 | 2.55 | ± | 0.58 | 0.52 | ± | 0.07 | 7.66 | ± | 0.01 | 0.06 | ± | 0.01 | 3.71 | ± | 0.13 | 11.93 |
| SB3 | 3rd | 1.03 | ± | 0.19 | 7.51 | ± | 0.32 | 8.44 | ± | 1.81 | 17.22 | ± | 0.62 | 5.70 | ± | 0.21 | 6.00 | ± | 1.34 | 0.70 | ± | 0.08 | 14.73 | ± | 0.56 | 0.06 | ± | 0.01 | 2.96 | ± | 0.01 | 22.04 |
| SB4 | 1st | 0.09 | ± | 0.02 | 6.20 | ± | 0.62 | 3.64 | ± | 0.58 | 17.24 | ± | 0.47 | 5.65 | ± | 0.35 | 4.79 | ± | 0.58 | 0.38 | ± | 0.06 | 24.67 | ± | 0.37 | 0.11 | ± | 0.01 | 2.10 | ± | 0.01 | 13.28 |
| SB4 | 2nd | 0.16 | ± | 0.03 | 6.45 | ± | 0.60 | 3.62 | ± | 0.36 | 17.78 | ± | 0.40 | 5.45 | ± | 0.37 | 2.46 | ± | 0.63 | 0.60 | ± | 0.15 | 8.71 | ± | 0.19 | 0.02 | ± | 0.01 | 4.66 | ± | 0.01 | 20.44 |
| SB4 | 3rd | 2.74 | ± | 0.76 | 6.75 | ± | 0.52 | 7.69 | ± | 1.32 | 17.18 | ± | 0.64 | 5.83 | ± | 0.18 | 3.65 | ± | 0.85 | 1.65 | ± | 0.23 | 14.20 | ± | 0.37 | 0.02 | ± | 0.01 | 7.32 | ± | 0.13 | 20.64 |
