# Supplementary material for: The Effects of Spatial Scale on Breakdown of Leaves in a Tropical Watershed
Source: PLoS One. 2014 May 8;9(5):e97072. doi: 10.1371/journal.pone.0097072 (PMC4014586; doi:10.1371/journal.pone.0097072)
Supplement: Figure S1 — Remaining mass over time in sampling sites. Percentages of remaining mass along of the day in E. cloeziana (A and C) and I. laurina (B and D), between stream order (A e B) and sub-basin (C and D). (DOCX) [file pone.0097072.s002.docx]

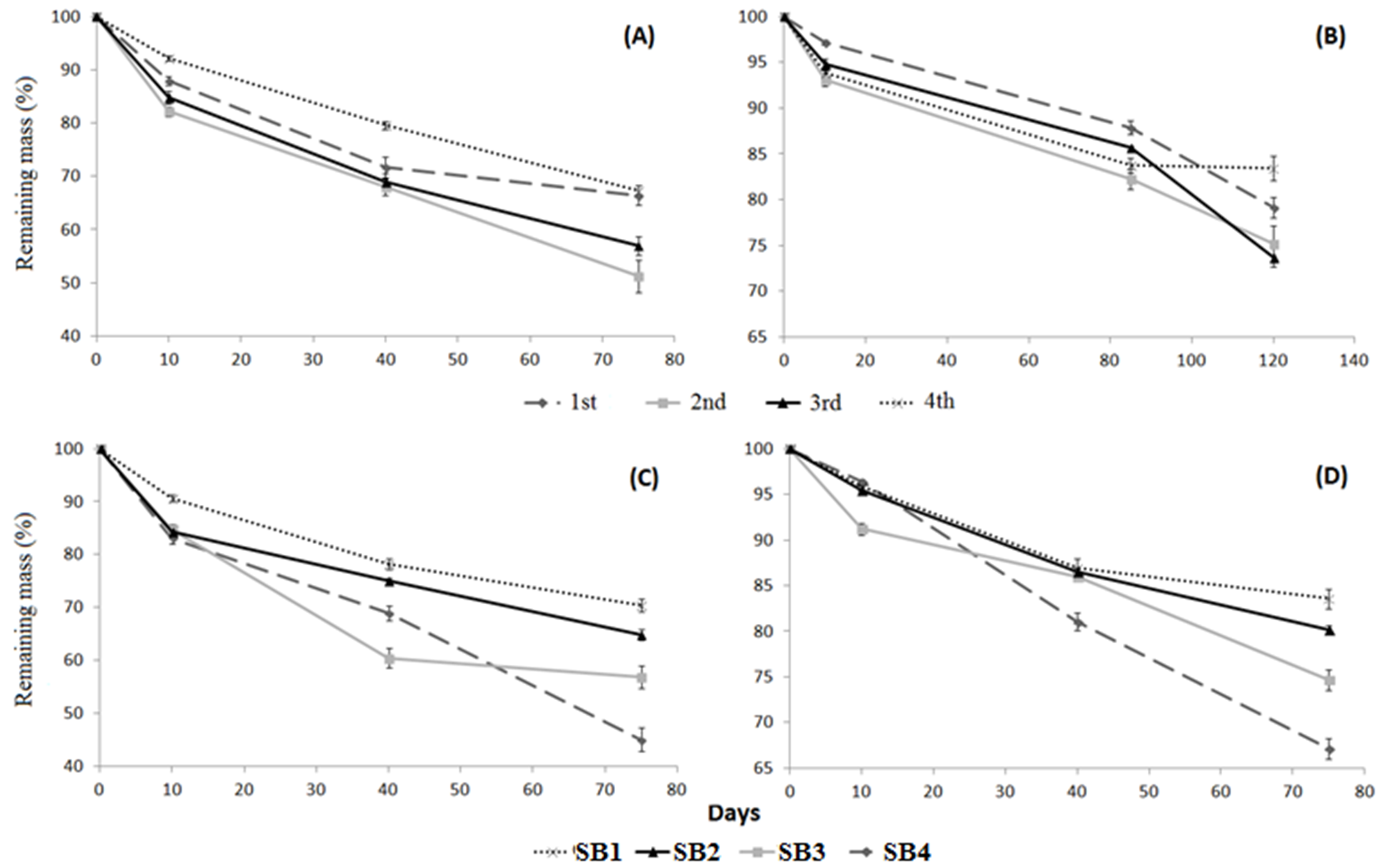


Figure S2. Percentages of remaining mass along of the day in *E. cloeziana* (A and C) and *I. laurina* (B and D), between stream order (A e B) and sub-basin (C and D).
