# Supplementary material for: The Effects of Spatial Scale on Breakdown of Leaves in a Tropical Watershed
Source: PLoS One. 2014 May 8;9(5):e97072. doi: 10.1371/journal.pone.0097072 (PMC4014586; doi:10.1371/journal.pone.0097072)
Supplement: Figure S2 — Biotic Community over time in E. cloeziana detritus. Average values and standard error of density (A and B), richness (C and D), biomass (E and F) of aquatic invertebrates, total microbial biomass (ATP; G and H) and fungal hyphomicetos biomass (I and J) along of the days in E. cloeziana, among stream order (A, C, E, G and I) and sub-basin (B, D, F, H and J). (DOCX) [file pone.0097072.s003.docx]

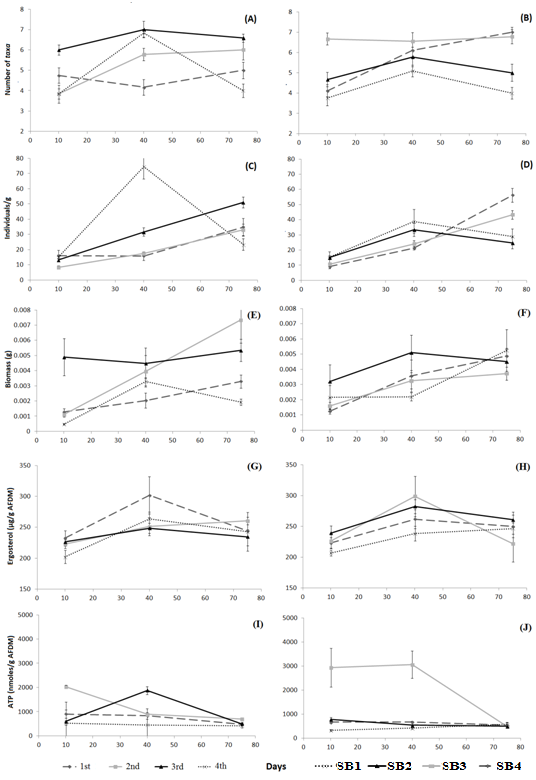


Figure S3. Average values ​​and standard error of density (A and B), richness (C and D), biomass (E and F) of aquatic invertebrates, total microbial biomass (ATP; G and H) and fungal hyphomicetos biomass (I and J) along of the days in *E. cloeziana,* among stream order (A, C, E, G and I) and sub-basin (B, D, F, H and J).
